# Quantitative modeling and analytic assessment of the transcription dynamics of the XlnR regulon in *Aspergillus niger*

**DOI:** 10.1186/s12918-016-0257-4

**Published:** 2016-01-29

**Authors:** Jimmy Omony, Astrid R. Mach-Aigner, Gerrit van Straten, Anton J.B. van Boxtel

**Affiliations:** Molecular Genetics Department, Groningen Biomolecular Sciences and Biotechnology Institute, University of Groningen, Nijenborgh 7, Groningen, 9747 AG The Netherlands; TU Wien, Institute of Chemical Engineering, Gumpendorfer Str. 1a, Wien, A-1060 Austria; Biobased Chemistry and Technology, Wageningen University, P.O. Box 17, Wageningen, 6700 AA The Netherlands

**Keywords:** Dynamic modeling, Parameter estimation, XlnR regulon, *Aspergillus niger*, D-xylose, CreA

## Abstract

**Background:**

Transcription of genes coding for xylanolytic and cellulolytic enzymes in *Aspergillus niger* is controlled by the transactivator XlnR. In this work we analyse and model the transcription dynamics in the XlnR regulon from time-course data of the messenger RNA levels for some XlnR target genes, obtained by reverse transcription quantitative PCR (RT-qPCR). Induction of transcription was achieved using low (1 mM) and high (50 mM) concentrations of D-xylose (Xyl). We investigated the wild type strain (Wt) and a mutant strain with partial loss-of-function of the carbon catabolite repressor CreA (Mt)*.*

**Results:**

An improved kinetic differential equation model based on two antagonistic Hill functions was proposed, and fitted to the time-course RT-qPCR data from the Wt and the Mt by numerical optimization of the parameters. We show that perturbing the XlnR regulon with Xyl in low and high concentrations results in different expression levels and transcription dynamics of the target genes. At least four distinct transcription profiles were observed, particularly for the usage of 50 mM Xyl. Higher transcript levels were observed for some genes after induction with 1 mM rather than 50 mM Xyl, especially in the Mt. Grouping the expression profiles of the investigated genes has improved our understanding of induction by Xyl and the according regulatory role of CreA.

**Conclusions:**

The model explains for the higher expression levels at 1 mM versus 50 mM in both Wt and Mt. It does not yet fully encapsulate the effect of partial loss-of-function of CreA in the Mt. The model describes the dynamics in most of the data and elucidates the time-dynamics of the two major regulatory mechanisms: i) the activation by XlnR, and ii) the carbon catabolite repression by CreA.

**Electronic supplementary material:**

The online version of this article (doi:10.1186/s12918-016-0257-4) contains supplementary material, which is available to authorized users.

## Background

The fungus *Aspergillus niger* has numerous economic and ecological applications, for example in paper manufacturing, animal feed and human foods. It is used in industrial fermentation processes for the production of organic acids like citric acid [1, 2], gluconic acid [3] and also for the production of enzymes such as amylase, amyloglucosidase, cellulases, pectinases, lactase, invertase and acid proteases in the food industry [4, 5]. Biotechnological products from *A. niger* are applied in the pharmaceutical, cosmetic and chemical industries [6]. Understanding gene transcription (mRNA synthesis) is crucial for advancing industrial applications and controlling the synthesis of target compounds from *A. niger*. Transcription of genes coding for xylanolytic and cellulolytic enzymes in *A. niger* is controlled by the transactivator XlnR [7]. XlnR plays a major role in regulating the expression of plant cell wall degrading enzymes (PCWDE) [8–10]. The target genes of the XlnR regulon encode amongst others the main xylanolytic enzymes xylanases B and C and β-xylosidase, and accessory enzymes like α-glucuronidase A, acetylxylan esterase A, arabinoxylan arabinofuranohydrolase A, and feruloyl esterase A [11]. The XlnR regulon also comprises genes coding for cellulolytic enzymes like endo-glucanases A, B, C and cellobiohydrolases A and B.

Carbon catabolite repression is a regulatory mechanism occurring in microorganisms leading to the adjustment of their carbon metabolism, thereby minimizing energy demands. The carbon catabolite repressor protein CreA was initially described in *Aspergillus nidulans* [12]. In the presence of high concentrations of D-xylose (Xyl), CreA modulates XlnR-induced expression of the genes involved in xylan degradation [13]. Up-regulation of several PCWDE in *A. niger* can result from carbon starvation or *creA* deletion [10, 14]. Mach-Aigner et al. [15] assessed the effect of CreA on transcription of PCWDE-encoding genes in *A. niger* by comparing the response to Xyl in two strains: the wild type (Wt) and a CreA mutant strain (Mt). They analyzed the transcript levels of genes coding for PCWDE, evaluated their Xyl concentration dependent expression profiles and described the role of CreA and XlnR in regulating transcription in *A. niger*. They found that genes coding for enzymes with similar function responded in a similar manner to a particular Xyl concentration and noted that utilization of high Xyl concentration is beneficial for the induction of hemicellulase-encoding genes [15].

Regulatory network reconstruction involves the analysis of trends and dynamics in data, and the inference of functional and regulatory mechanisms in biological systems. Mathematical modeling helps us to represent and understand complex interactions in networks. Modeling enables prediction of the resultant effect of nonlinear interactions in a network [16], thereby providing insight into cellular regulatory processes and kinetics [17]. Ordinary differential equations (ODEs) are a popular formalism for modeling time responses in regulatory networks [18–23]. ODEs used in the context of modeling biological networks consist of mechanistic representations of the transcription rates in biological (sub-)systems [19]. Dynamic modeling involves incorporating prior knowledge into models of the regulatory network.

Omony et al. [24] provided a dynamic model based on ODE for the description of the regulation mechanisms and dynamics of the XlnR regulon. Mach-Aigner et al. [15] investigated the role of CreA using time course data (TCD) based on an hourly sampling interval. However, the information in this time interval was not detailed enough to adapt the previous dynamic model [24]. The purpose of the current work is to make amendments to the model based on experimental data from shorter time intervals, that better resolve the transcript dynamics. It also allows for a variety of regulation mechanisms for the XlnR target genes, rather than a single mechanism as proposed in previous models [24]. Additionally, by comparing the Wt with Mt data, an attempt is made to obtain more insight in the mechanism underlying the CreA regulatory influence.

## Methods

### Strains, growth conditions, RT-qPCR

The strains (*A. niger* N400 (CBS120.49) (Wt) and the CreA mutant strain NW283 that exhibits a de-repressed phenotype (Mt)), growth conditions, RNA-extraction, reverse transcription and the quantitative PCR analyses in this work were described by Mach-Aigner et al. [15]. TCD were obtained for 23 genes for the Wt using a sampling interval of 20 minutes during a period of 5 hours, and for 8 genes for the Mt using an hourly sampling interval (see Additional files 1 and 2). Strains were grown in bioreactors on sorbitol, which was used as a non-inducing/non-repressing carbon source. The experiments involved quantifying expression of: *xlnR*, genes encoding endoxylanases (*xlnB* and *xlnC*), β-xylosidase (*xlnD*), arabinoxylan arabinofuranohydrolase (*axhA*), acetylxylan esterase (*axeA*), α-glucuronidase (*aguA*), feruloyl esterase (*faeA*), endoglucanase *eglA* and *eglB,* which are XlnR target genes [25]. The other XlnR target genes are: *eglC*, *talB*, *xdhA*, *ladA, estA* and the Xyl reductase-encoding gene *xyrA* as well as *abfB*, *bglA* and *xkiA* [26]. XlnR also regulates expression of α- and β-galactosidase-encoding genes (*aglB* and *lacA*) [27] and cellobiohydrolase-encoding genes *cbhA* and *cbhB* [28].

### Previous model formulation for the XlnR regulon dynamics

Based on prior knowledge of the regulatory mechanisms of the XlnR regulon described by de Vries et al. [13], the original model of Omony et al. [29] was formulated as follows:

1 $$ \left\{\begin{array}{c}\hfill {\overset{.}{x}}_{xlnR}=bu-{k}_d{x}_{xlnR}\hfill \\ {}\hfill {\overset{.}{x}}_i={k}_{is}\frac{{\left({k}_{i1}{x}_{xlnR}\right)}^{h_1}}{1+{\left({k}_{i1}{x}_{xlnR}\right)}^{h_1}}\frac{1}{1+{\left({k}_{i2}u\right)}^{h_2}}-{k}_{id}{x}_i\hfill \\ {}\hfill \mathrm{given}\ {x}_{xlnR}(0),\ {x}_i(0),\ u(0)\hfill \end{array}\right. $$

where *x*_*xlnR*_ - activity state for *xlnR*, *u* - Xyl concentration, *b* - input stimulus coefficient, *k*_*i*2_ - inverse of Hill constant for CreA, *k*_*d*_ - mRNA degradation parameter for *xlnR*, *x*_*i*_ - activity state for target gene *i* and ${\overset{.}{x}}_i$ – transcription rate. The transcription rate is constant when the gene is on, but is reduced by the two switching Hill functions in the first term of the right hand side, one for activation by XlnR and the other for stimulus proportional de-repression.

Omony et al. [29] used Hill coefficients *h*_1_ = *h*_2_ = 1. The *h*_1_ values govern the transcriptional response of target genes to Xyl induction. A small value, *h*_1_ ≈ 1 may lead to a graded response, whereas larger values are more likely to result in a bi-stable switch-like response in gene expression profiles [30]. Ninfa and Mayo [31] established a positive association between high Hill coefficients and transcription dynamics. Many biological systems have large Hill coefficients (*h*_*l* = 1,2_ ≥ 2) [32] which are associated with multi-stability, higher order dynamics, and the number and specificity of transcription factor (TF) binding sites. Our data suggest that steeper switching is more appropriate; therefore, in this work we used higher *h*_1_ and *h*_2_ values. We considered Xyl to cooperatively interact with XlnR at the promoter site of target genes, as opposed to earlier proposed binding mechanisms [29].

### New model formulation

To describe the regulatory mechanism of the dependence of the XlnR regulon on Xyl and CreA, we define the *xlnR* promoter as *P*_*xlnR*_. The normalized transcript levels indicate that the expression of *xlnR* remains nearly unchanged (values ~1 to 2 relative to the *hist* gene), irrespective of the used Xyl concentration (Figs. 2H; 3H; 4 W and 5 W). In the model, the resultant protein (XlnR) is considered to ultimately bind cooperatively with Xyl to the promoter of a target gene forming the complex *x*_*xlnR*_*u*. The concentration of this complex, which is also considered as the active form of XlnR, is denoted by *X* = [*x*_*xlnR*_*u*]. This complex dissociates into [*x*_*xlnR*_] and [*u*], which hereafter are simply denoted by *x*_*xlnR*_ and *u*, respectively. CreA binds to the promoters of the target genes, thus inhibiting transcription. The number of CreA binding sites varies between the promoters of the target genes, however, we assume that occupation of at least a single binding site suffices to repress transcription.

**Fig. 1:**
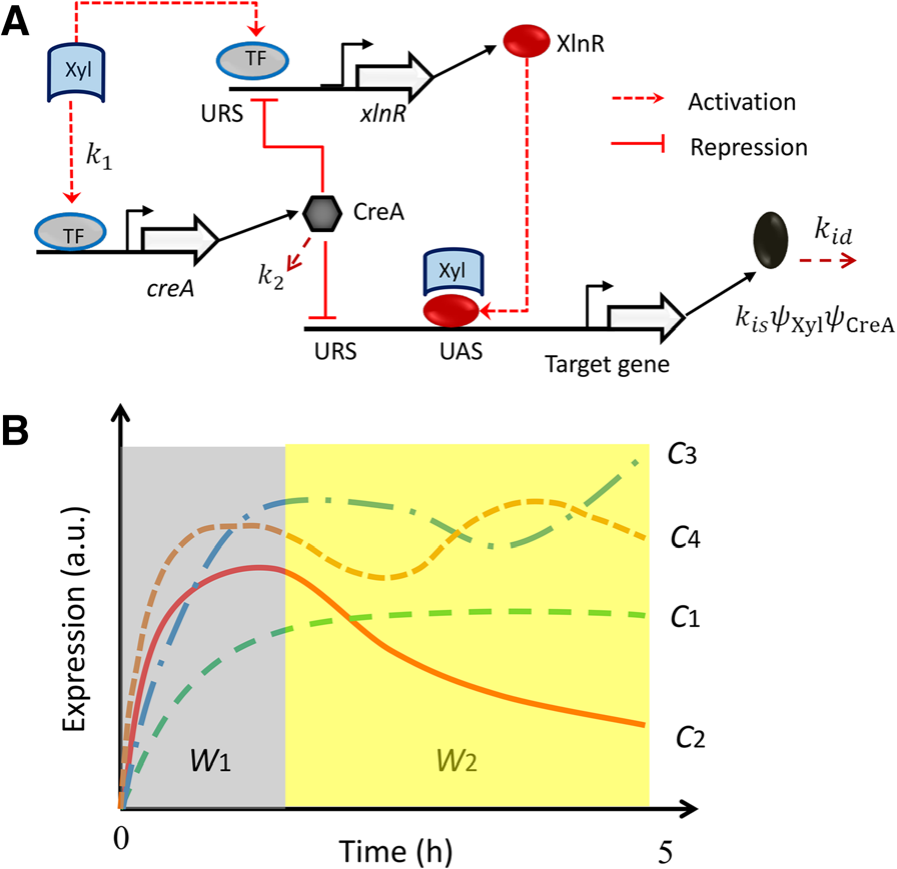
Regulatory mechanisms in the XlnR regulon and transcription profile classification. **a**: Model of how XlnR and CreA control transcription of the XlnR target genes. The term TF represents a transcription factor. URS and UAS – upstream repression and activation sequences, respectively. The depicted CreA repression of *xlnR* is hypothetical. **b**: The transcription dynamics were classified as: *C*_1_ – a monotonic increasing function, *C*_2_ – a function with a maximum and a lower steady state, *C*_3_ – a function that steadily rises to a maxima, decreases and then again increases (de-repression of transcription), and *C*_4_ – bimodal expression with de-repression. *W*_1_ and *W*_2_ are time window partitions. The term a.u. refers to arbitrary units. Any other parameters are as described in the materials and methods section

**Fig. 2:**
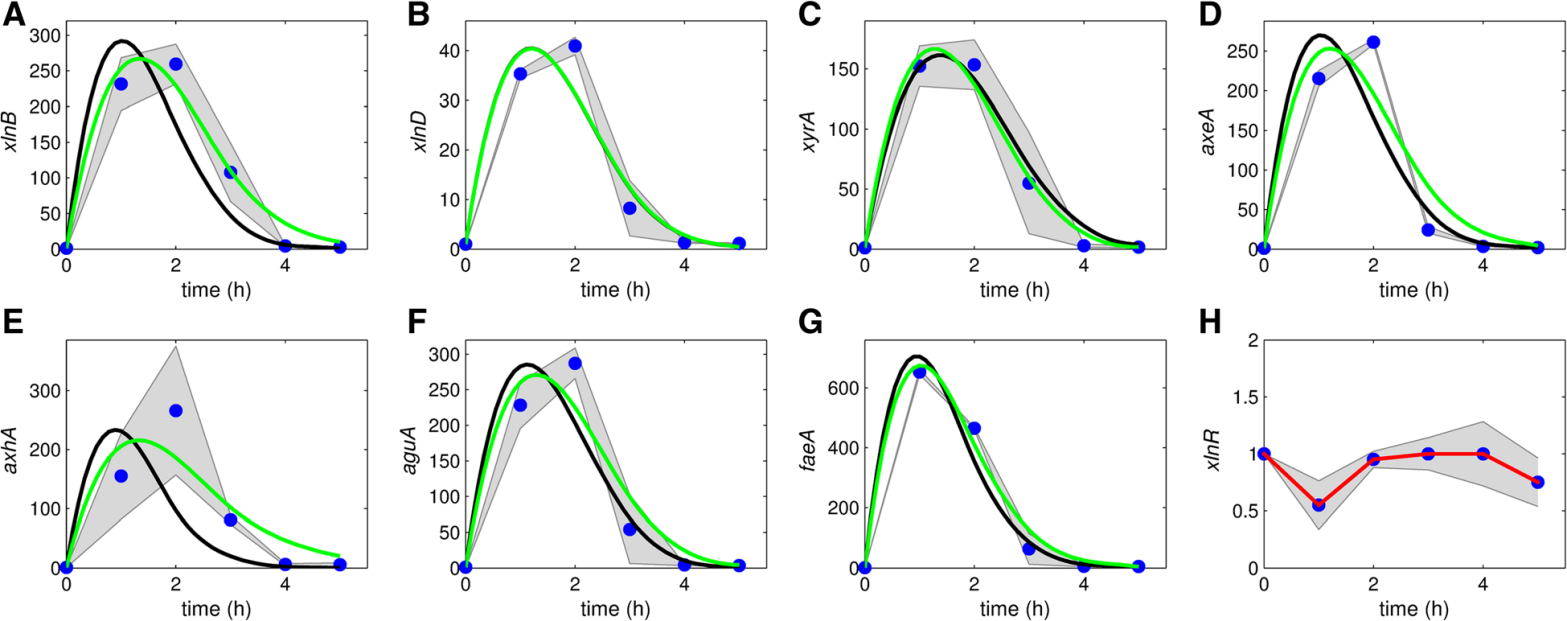
Model fit to the data obtained for the Mt using 1 mM Xyl. **a**-**g**: Black bold line is the model fit to data with all parameters fixed except *k*_*is*,Mt_ and *K*_*i*2,Mt_. The green bold line is the fit with all parameters fixed except *k*_*is*,Mt_, *K*_*i*2,Mt_ and *k*_*id*_. ${\widehat{\overline{K}}}_{\mathrm{on}}=14.957$, ${\widehat{\overline{K}}}_{\mathrm{off}}=75.541$, ${\widehat{\overline{k}}}_1=21.455$ and ${\widehat{\overline{k}}}_2=20.065$ were fixed. The graphs are plotted on different scales to aid visibility and the shaded areas indicate standard deviations. **h**: transcript levels of *xlnR*. The red line is the fit to the mean *xlnR* expression values. The dots represent normalized and averaged RT-qPCR data

**Fig. 3:**
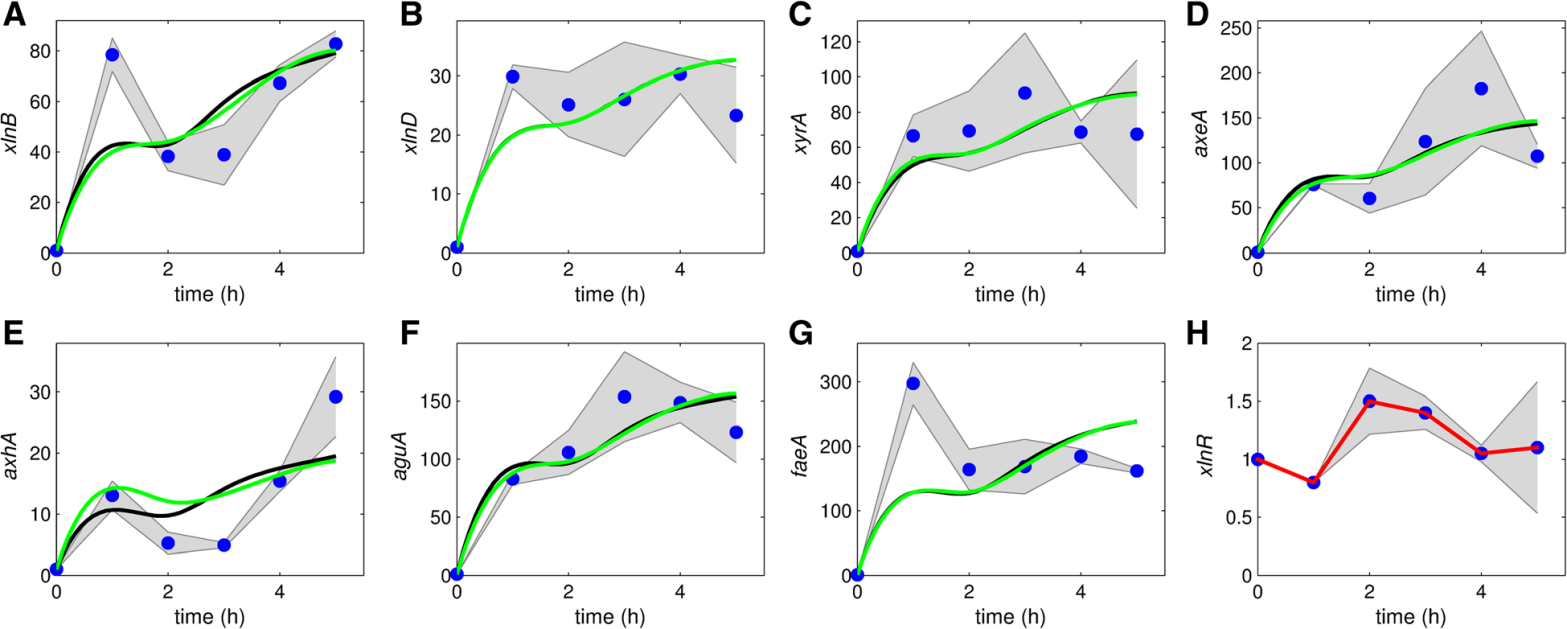
Model fit to the data obtained for the Mt using 50 mM Xyl. **a**-**g**: The black bold line is the model fit to data with all parameters fixed except *k*_*is*,Mt_ and *K*_*i*2,Mt_. The green bold line is the fit with all parameters fixed except *k*_*is*,Mt_, *K*_*i*2,Mt_ and *k*_*id*_. ${\widehat{\overline{K}}}_{\mathrm{on}}=14.957$, ${\widehat{\overline{K}}}_{\mathrm{off}}=75.541$, ${\widehat{\overline{k}}}_1=21.455$ and ${\widehat{\overline{k}}}_2=20.065$ were fixed. The graphs are plotted on different scales to aid visibility and the shaded areas indicate standard deviations. **h**: *xlnR* transcript levels. The red line is the fit to the mean *xlnR* expression values. The blue dots represent normalized and averaged RT-qPCR data

**Fig. 4:**
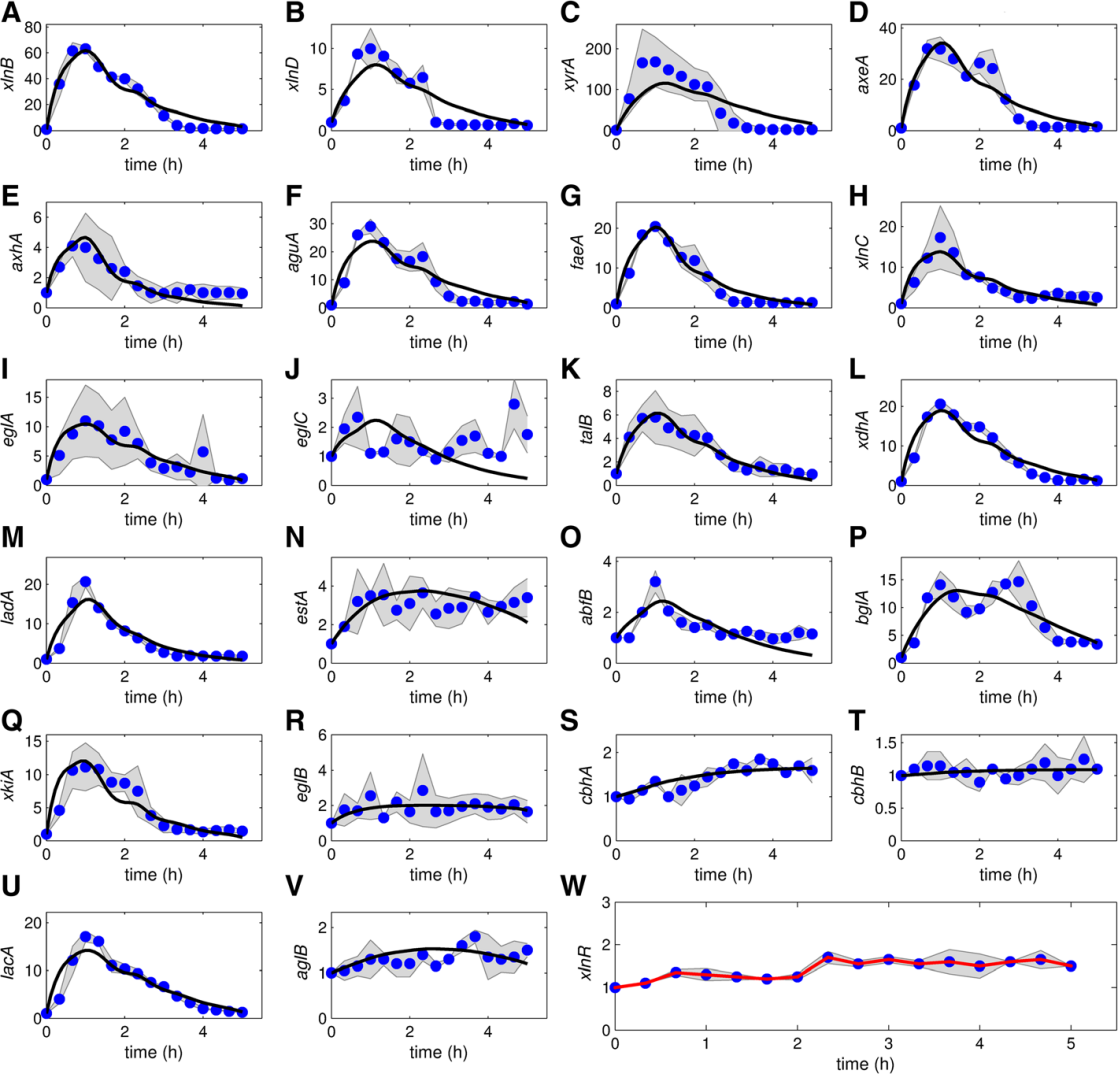
Model fit to the TCD obtained for the Wt using 1 mM Xyl. **a**-**v**: ${\widehat{\overline{K}}}_{\mathrm{on}}=14.957$, ${\widehat{\overline{K}}}_{\mathrm{off}}=75.541$, ${\widehat{\overline{k}}}_1=21.455$ and ${\widehat{\overline{k}}}_2=20.065$ were fixed. **w**: *xlnR* transcript levels. The red line is the model fit to the mean *xlnR* expression values. The graphs are plotted on different scales to aid visibility and the shaded areas indicate standard deviations. The blue dots represent normalized and averaged RT-qPCR data. The black lines show the model fit to the data points

**Fig. 5:**
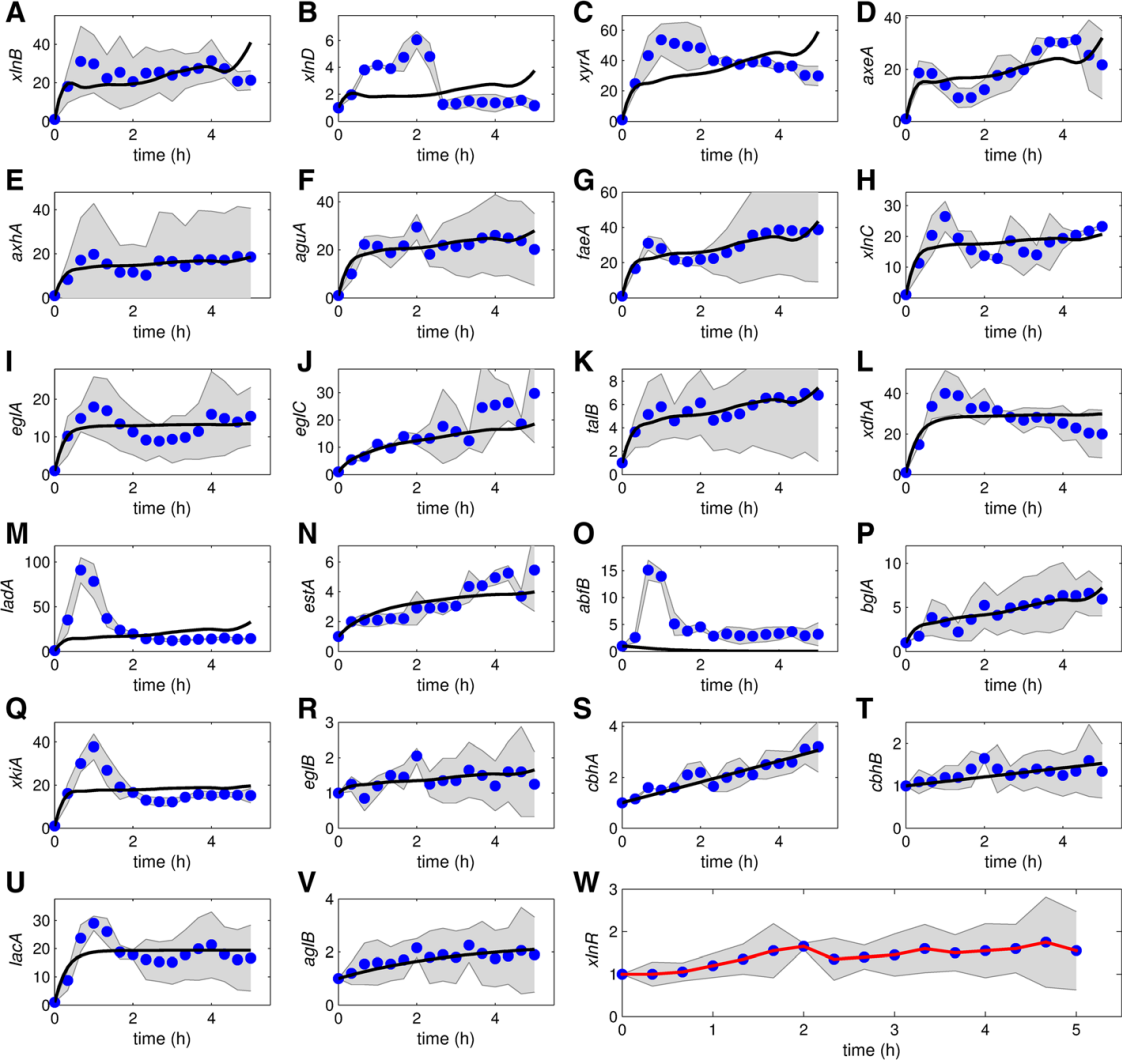
Model fit to the TCD obtained for the Wt using 50 mM Xyl. **a**-**v**: ${\widehat{\overline{K}}}_{\mathrm{on}}=14.957$, ${\widehat{\overline{K}}}_{\mathrm{off}}=75.541$, ${\widehat{\overline{k}}}_1=21.455$ and ${\widehat{\overline{k}}}_2=20.065$ were fixed. **w**: *xlnR* transcript levels. The red line is the fit to the mean *xlnR* expression values. The graphs are plotted on different scales to aid visibility and the shaded areas indicate standard deviations. The blue dots represent normalized and averaged RT-qPCR data. The black lines show the model fit to the data points

*xlnR* is constitutively transcribed, irrespective of the Xyl concentration [15]. It is evident from our data (Figs. 2, 3, 4 and 5) that the pseudo-steady state assumption ${\tilde{x}}_{xlnR}=b\tilde{u}/{k}_d$ based on earlier proposed models for the expression of *xlnR* [29] cannot be maintained. Therefore, an improved model based on the mechanism in Fig. 1A is used to explain the network dynamics. The regulation mechanism for the XlnR regulon is modeled using the equations

2 $$ \overset{.}{X}={K}_{\mathrm{on}}{x}_{xlnR}u-{K}_{\mathrm{off}}X $$

3 $$ {\overset{.}{x}}_{\mathrm{CreA}}={k}_1u-{k}_2{x}_{\mathrm{CreA}} $$

4 $$ {\overset{.}{x}}_i={k}_{is}\left(\frac{X^{h_1}}{K_{i1}^{h_1}+{X}^{h_1}}\right)\left(\frac{K_{i2}^{h_2}}{K_{i2}^{h_2}+{x_{\mathrm{CreA}}}^{h_2}}\right)-{k}_{id}{x}_i $$

given [*X*(0), *x*_CreA_(0), *x*_*i*_(0)]^*T*^; *k*_*is*_ – mRNA synthesis parameter, *k*_*is*_ ∈ {*k*_*is*,Wt_, *k*_*is*,Mt_} – maximum transcription rate in the Wt and Mt, respectively. The state variables depict mRNA concentration, protein abundance and other time-dependent quantities.

Here *K*_on_ (h^-1^) and *K*_off_ (h^-1^) are the association and dissociation constants, respectively. *K*_on_ describes the interaction between XlnR and Xyl, and *K*_off_ the strength of the interaction between the two molecules. *x*_CreA_ depicts CreA concentration, *k*_1_ and *k*_2_ are constants. *K*_*i*1_ is the expression threshold parameter (half-saturation concentration) that corresponds to activation of target gene *i* by the complex of XlnR and Xyl. *K*_*i*2_ ∈ {*K*_*i*2,Wt_, *K*_*i*2,Mt_} is an expression threshold parameter (half-saturation concentration). It corresponds to CreA repression in the Wt and Mt, respectively. This allows for a partial or complete CreA loss-of-function Mt to be accounted for in the model. *k*_*id*_ is the first-order rate constant for the mRNA degradation for target gene *i*.

Let ${\psi}_{\mathrm{CreA}}={K}_{i2}^{h_2}/\left({K}_{i2}^{h_2}+{x_{\mathrm{CreA}}}^{h_2}\right)$ be the Hill function associated with CreA repression. Alternatively, it can be written as ${\psi}_{\mathrm{CreA}}=1/\left(1+{\left({x}_{\mathrm{CreA}}/{K}_{i2}\right)}^{h_2}\right)$, which illucidates on the switiching level *K*_*i*2_ and steepness parameter *h*_2_. For partial CreA repression, *x*_CreA_ ≠ 0, with 0 < *ψ*_CreA_ < 1. The value *h*_2_ ≫ 1 represents increasingly steeper switching around the switching level *K*_*i*2_. We used *h*_2_ = 4 throughout for the modeling. CreA mutation implies that *x*_CreA_ ≈ 0, therefore, *ψ*_CreA_ ≈ 1 in equation (4), in which case equation (3) is not required. Alternatively, a partial loss-of-function of CreA can be represented by maintaining equation (3), but adjusting *K*_*i*2_ and/or *h*_2_ (in case of less effective binding sites). We define ${\psi}_{\mathrm{Xyl}}={X}^{h_1}/\left({K}_{i1}^{h_1}+{X}^{h_1}\right)$ as the Hill function associated to the active form of XlnR, which according to equation (1) is associated to Xyl and *xlnR*. In the modeling, the basal transcription level for each transcript is considered as negligible compared to the actual transcript levels.

The parameters *K*_*i*1_ and *K*_*i*2,{Wt|Mt}_ are related to the chemical affinity between the respective TFs and their binding sites [33]. The model structure is the same for the XlnR targets, except for variations in the levels of CreA repression. The parameters *k*_*is*,Mt_ and *k*_*is*,Wt_ are used to assess the mRNA levels for gene *i* in the Mt and Wt. Let *λ*_*i*_ := *k*_*is*,Mt_/*k*_*is*,Wt_ be the proportional fold-change between the mRNA level in the Mt to the Wt. It is likely that *λ*_*i*_ ≫ 1 for most target genes, since higher transcript levels were observed in the Mt than Wt. Transcription in the Mt does not significantly differ from the Wt when *λ*_*i*_ ≈ 1. Overall, we tested the hypotheses that: (i) expression of the XlnR targets is not regulated by CreA repression, (ii) not all response patterns *C*_3_ - *C*_4_ (described below) can be generated by the model in equations (2) to (4).

In equations (2) to (4) we assumed: (a) Xyl cooperatively binds with XlnR at *P*_*xlnR*_ and CreA binds to the DNA of XlnR targets. The new model formulation allows us to explain higher order dynamics by assuming, (b) negligible time-delay in translation, (c) transcription only occurs if at least a TF binding site is occupied, (d) all transcribed genes are translated into proteins, (e) all TFs bind independently to the target gene promoter, and (f) mRNA and protein degradation is not regulated.

### Fitting the model to the data

The model fit to data was conducted separately and sequentially, starting with the Wt followed by the Mt. In each strain, the model was simultaneously fitted to the data corresponding to use of 1 and 50 mM Xyl. We used the sum of squared errors minimization between the measured and estimated data values. The combined goal function is the sum of two functions one belonging to each of the 1 and 50 mM Xyl induction. Equations (2) to (4) were fitted to the data resulting in a single vector of parameter estimates, *θ*_*i*_ where

5 $$ {\theta}_i={\left[{K}_{\mathrm{on}},{K}_{\mathrm{off}},{k}_1,{k}_2,{k}_{is},{K}_{i1},{K}_{i2},{k}_{id}\right]}^T $$

where *θ*_*i*_ is a concatenation of vectors *ϑ*_1_ = [*K*_on_, *K*_off_, *k*_1_, *k*_2_]^*T*^ and *ϑ*_2_ ∈ {*ϑ*_2,Wt_, *ϑ*_2,Mt_}; *ϑ*_2,Wt_ = [*k*_*is*,Wt_, *K*_*i*1_, *K*_*i*2,Wt_, *k*_*id*_]^*T*^ and *ϑ*_2,Mt_ = [*k*_*is*,Mt_, *K*_*i*1_, *K*_*i*2,Mt_, *k*_*id*_]^*T*^; *K*_*i*1_ and *k*_*id*_ are considered as the same in the Wt and Mt. These two parameters were later fixed for the estimation of *k*_*is*,Mt_ and *K*_*i*2,Mt_ using the Mt datasets. In equation (5), *θ*_*i*_ ∈ {*θ*_*i*,Wt_, *θ*_2,Mt_}, where *θ*_*i*,Wt_ = [*ϑ*_1_, *ϑ*_2,Wt_]^*T*^ and *θ*_*i*,Mt_ = [*ϑ*_1_, *ϑ*_2,Mt_]^*T*^ for the Wt and Mt, respectively. The optimization routine consisted of the goal functions: *J*_*i*,1_(*θ*_*i*_) = *min*∑_*j* = 0_^*N*^(*y*_*ij*_ − *ŷ*_*ij*_)^2^ and *J*_*i*,50_(*θ*_*i*_) = *min*∑_*j* = 0_^*N*^(*y*_*ij*_ − *ŷ*_*ij*_)^2^ for the 1 and 50 mM induction dataset, respectively. The terms *y*_*ij*_ and *ŷ*_*ij*_ respectively refer to the measured and estimated data value for gene *i* at time instant *j*.

The model fit to data was performed one gene at a time. The goal functions were combined according to the expression *J*_comb_(*θ*_*i*_) = *J*_*i*,1_(*θ*_*i*_) + *J*_*i*,50_(*θ*_*i*_). The optimization was performed using the *lsqnonlin* routine in MathWorks Matlab R2013a. The results for ${\widehat{\theta}}_i$ are given in (Additional file 3: Table S1 and S2). Initially, ${\widehat{\theta}}_i$ was obtained in the Wt for all genes and the averages of some parameters were fixed at ${\widehat{\overline{\vartheta}}}_1$ and the remaining *ϑ*_2_ re-estimated. Here ${\widehat{\overline{\vartheta}}}_1$ is the vector of parameter estimates averaged (and fixed) for all target genes. It contains the least sensitive parameters in the models. On the contrary, ${\widehat{\vartheta}}_2$ contains estimates for non-fixed parameters, which are more sensitive than those in ${\widehat{\overline{\vartheta}}}_1$. The coefficient of variation (CV) is defined as ${\mathrm{CV}}_{{\widehat{\vartheta}}_2}=\mathrm{S}\mathrm{D}\left({\widehat{\vartheta}}_2\right)/{\widehat{\vartheta}}_2$ where SD is the standard deviation.

## Results

### Qualitative model hypothesis based on analysis of the data

Overall, four classes of transcription patterns were observed in our data (Fig. 1b, classes *C*_1_ - *C*_4_). *C*_1_ is a monotonic increasing function; transcription under *C*_2_ reaches a maximum value after which it gradually decreases to a new steady state. In *C*_3_ and *C*_4_ there is strong evidence of de-repression (time window *W*_2_, Fig. 1b). The models proposed in this study describe the transcription dynamics for some target genes, especially those belonging to *C*_1_. In *C*_2_, first, transcription is activated by Xyl, it attains a maximum value and then decreases. This decrease corresponds to Xyl depletion. The transcription dynamics for some genes could not be captured by our models, e.g. *abfB,* which has no binding sequence for XlnR [25]; hence, we would conclude that it is not part of the XlnR regulon.

Using our observations and the regulatory model for the XlnR targets as shown in the equations and Fig. 1a, the behavior can be qualitatively described as follows. Initially, transcription is triggered by Xyl-mediated induction (insignificant repression of transcription). At this instance, the CreA concentration is low but gradually builds up to eventually repress transcription (Fig. 1a), as long as Xyl is present in the medium. Transcription is reduced again when Xyl gets depleted, as is depicted in class *C*_2_. The conceptual scheme allows differences in gene expression between various target genes by varying the degree of induction or repression for each individual gene.

### The XlnR regulon transcription dynamics

An initial experiment was performed using the Mt and analyzing 7 target genes of the XlnR regulon, in order to evaluate manageable sampling intervals and to obtain insight into Xyl utilization. In a second, more extensive experiment, the Wt was used and more detailed TCD for 23 genes could be obtained. We observed that: (i) XlnR is constitutively expressed, (ii) the target gene expression levels reach higher values at a stimulus of 1 mM compared to 50 mM, both in the Wt as in the Mt, and (iii) in the Mt target gene expression levels are generally higher than in the Wt (Figs. 2, 3, 4 and 5). Expression of *xlnR* in the Mt was marginaly less than in the Wt at most time points (Figs. 2 and 3H; 4 and 5 W).

A similar up-regulation dynamic of the XlnR target genes occur within the first hour after Xyl induction (Figs. 2 and 3); however, after 1 h, the same genes show varying patterns in transcription dynamic and the intensity of gene expression (Fig. 4). This difference may partly be attributed to a reduction in Xyl concentration. Unlike other target genes, transcription of e.g. *xlnD* and *bglA* is bimodal, i.e. they exhibit a repeated up and down pattern of expression (Fig. 4). Transcription values of some target genes (e.g. *eglB*, *cbhB* and *aglB;* Figs. 4 and 5: r, t and v) remained around their steady states, thus showing less interesting dynamics. Some genes exhibited dynamics that cannot be explained using both the previous and newly proposed models, especially using 50 mM Xyl (e.g. *xlnD*, *xyrA*, *axeA*, *ladA*, *abfB*, *xkiA* and *lacA*, Fig. 5).

Genes with similar or the same regulation mechanisms often have similar expression profiles and response to stimuli. Such genes are expected to cluster together in a dendogram. Dynamic time warping [34] was used to cluster the data, which enables identification of genes with similar in time-evolution profiles (Additional file 4: Figure S1). In the gene expression clusters, the placement for *axhA* differs in the Mt irrespective of the inducing Xyl concentration (Additional file 4: Figure S1A and B). In the Wt, induction with 1 mM Xyl resulted in 19 genes in a single cluster (Additional file 4: Figure S1C) while induction with 50 mM Xyl resulted in two large clusters each with 10 genes (Additional file 4: Figure S1D). Genes with bimodal transcription pattern like *xlnD* and *bglA* also cluster together in the case of both Xyl concentrations (Additional file 4: Figure S1C and D). Since the gene clusters are based on their transcription profiles, they intrinsically provide clues as to which genes might possess similar additional regulation mechanisms.

### Model results

After parameters estimation, our model fits quite well to most of the TCD (Figs. 2, 3, 4 and 5, black lines; Additional file 3: Table S1). Hence, it is possible to find parameter values that are consistent with the data, with the exception of some genes. For the 50 mM experiment the model fits very well to some of the gene profiles, e.g. *xyrA* and *axeA* and *aguA* in the case of the Mt (Fig. 3) and *xlnB*, *aguA* and *talB* in the case of the Wt (Fig. 5).

In the parameter estimation during model fitting to data, the covariance matrix had large correlation values between some parameters. Parameters with poorer estimates were fixed before rerunning the model fit; thereby, reducing the parameter degrees of freedom. To assess the influence of *k*_*id*_ , the models were fitted with and without fixing the parameters. Though not significantly pronounced, not fixing *k*_*id*_ results in a systematic shift of the model fit to the right (compare green line to the black line) for some genes in case of the Mt data obtained with 1 mM Xyl (Fig. 2). In case of the Mt data obtained with 50 mM Xyl not fixing *k*_*id*_ does not result in any significant difference in the model fit (compare green line to the black line) for all genes (Fig. 3). Overall, the difference between the model fits in green and black lines is insignificant.

It remains a challenge to accurately quantify CreA abundance. The lack of data on CreA results in some parameters being estimated with less precision (Additional file 3: Table S1). Such lack of data on relevant state variables still remains a huddle in modeling biological systems [35–37]. The number of parameters in equation (5) poses a challenge in the network inference because of the presence of correlated parameters. Another source of bias in parameter estimation is data measurement noise which complicates the surface of the objective function. This may introduce local minima in an already complex search space, especially for nonlinear models [38]. This need not be a major problem if the interest is to describe the transcription dynamics, but only when an attempt is made to attach a biological meaning to the estimated parameters. To unravel the XlnR regulon dynamics, the derived ${\widehat{\theta}}_i$ were used to analyze the Hill functions. The effects of the role interplay between *ψ*_Xyl_ and *ψ*_CreA_ can be seen from the variation in intensity of transcription on XlnR target genes (Additional file 5: Mt: Additional file 4: Figure S2, and S3A to G, Additional file 6: Wt: Additional file 4: Figure S4, and S5A to V), with possible antagonism between *ψ*_Xyl_ and *ψ*_CreA_.

Unlike induction with 50 mM Xyl (Additional file 4: Figure S3 and S5), the evaluation of the Hill function components using the parameter estimates shows that in case of induction with 1 mM Xyl, the effect of CreA is negligible, i.e. *ψ*_CreA_ ≈ 1 throughout the 5 h time-frame (compare Additional file 4: Figure S4). Otherwise, Xyl plays a major role (0 ≤ *ψ*_Xyl_ ≤ 1) in activating transcription (Additional file 4: Figure S2 and S4). This effect is generally reversed in the presence of high Xyl levels (Additional file 4: Figure S3 and S5). Unlike high Xyl concentration, for low Xyl induction the activation term *ψ*_Xyl_ and the mRNA degradation term *k*_*id*_*x*_*i*_ also plays a major role in determining the network dynamics; however, at high Xyl concentration the components *ψ*_Xyl_ and *ψ*_CreA_ jointly regulate transcription.

### The possibility of feedback by CreA

Additional parameter estimation exercises were done in an attempt to improve the fit, including the introduction of a feedback effect in the model (shown in Fig. 1a, but not in the equations). It was found that this did, indeed improve the fit to data for some genes, but also introduces bias in the parameter estimates. The data are not rich enough to estimate the feedback parameters with confidence. Including a feedback loop in the model requires prior knowledge of candidate molecules and their likely direction of impact, as a repressor or activator. Such a feedback effect may be further complicated if the molecules interact with other regulatory proteins that potentially also regulate the same target gene. Another reason for excluding the feedback effect in the models is because it would only further escalate the number of parameters required to describe the dynamics of the XlnR regulon; hence, the reason for using a relatively simple yet sufficiently powerful model to explain the observed transcription dynamics in the data. Therefore, the feedback mechanism was not further pursued in the current modeling.

## Discussion

It was not possible to obtain good fits by merely changing the switch level *K*_*i*2_ in the CreA inhibition function between Wt and Mt. Rather, the Wt and Mt switch levels remain roughly the same between the Wt and Mt. The estimated rate coefficient *k*_*is*_ indicates the relative quantity of mRNA molecules synthesized per unit time (Additional file 3: Table S1). Contrary to expectations the uninhibited transcription rate coefficient *k*_*is*_ must be set considerably higher in the Mt to obtain good fits. It is unclear which underlying mechanism in the Mt is responsible for the partial loss-of-function of CreA.

**Fig. 6:**
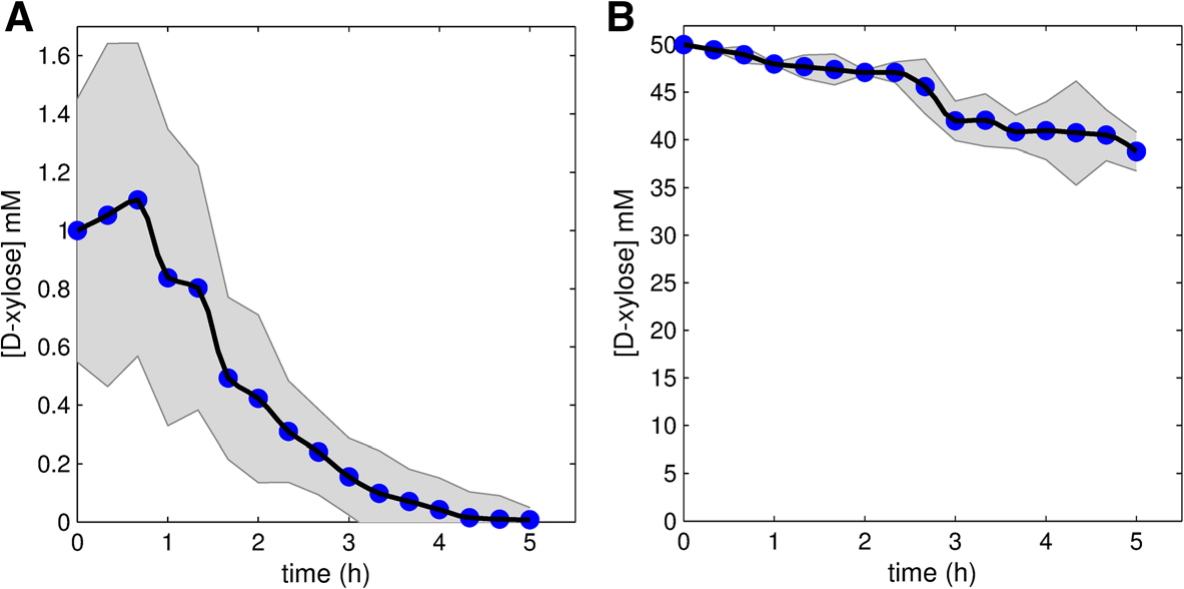
Xyl concentration. Concentration of Xyl during growth in the bioreactor was monitored by HPLC analyses. **a**: Induction was performed with 1 mM Xyl. **b**: Induction was performed with 50 mM Xyl. The shaded areas indicate standard deviations and the black lines indicate the mean fit through the data points

The transcription responses to the 50 mM Xyl induction are given in Figs. 3 and 5. Although the Xyl concentration decreases from 50 mM to ~38 mM (Fig. 6b) after around 5 h, the Xyl level is still high. About 80 % of the initial Xyl pulse is left by 5 h, indicating a gradual decrease in Xyl concentration. It was thus expected that the target gene response would follow a monotonic increasing curve, with a possible slow decrease due to decreasing Xyl concentration. The responses in Figs. 3 and 5 deviate significantly from this expectation.

Overall, some genes were lowly expressed and others had noisy expression profiles. Some genes were lowly expressed in the Wt, e.g. *eglB*, *cbhA*, *cbhB* and *aglB* in the case of both Xyl concentrations (Figs. 4 and 5). The gene *bglA* shows a bimodal transcription pattern with peaks at ~1 and ~3 h (Fig. 4P). The expression of *faeA* is controlled by CreA and XlnR, and it is also known to respond to other aromatic compounds [39]. To get insight into how the genes grouped in relation to similarity in their expression profiles, we performed a cluster analysis. Clustering of expression profiles (Additional file 4: Figure S1A, B and D) show that *faeA* falls in a unique group. A similar mechanism might exist for the other target genes, which would explain the complex dynamics in the data, particularly for induction with high Xyl concentration. Using 1 mM Xyl for induction, after 5 h nearly all Xyl is consumed (Fig. 6a).

ODEs can be used to describe regulatory mechanisms like the kinetics of protein-protein or protein-mRNA interactions. The dynamics in the gene expression profiles provide leads for testing candidate regulation mechanisms for the XlnR regulon. The response curves show the four types of behavior depicted in Fig. 1b, which are typical of high order dynamics, and show fast and slow mRNA degradation for some XlnR target genes. Transcription values of some target genes remained around their steady states and thus have less interesting dynamics. Many genes exhibited dynamics that fit our models (Figs. 2, 3, 4 and 5). The transcription profiles for some of the XlnR targets indicate the involvement of further regulatory mechanisms such as post-translational regulation. In such a case, we do not exclude the possibility that such regulatory mechanisms could be either single or multiple post-translational modification steps.

Even if the interaction between XlnR and Xyl was not yet experimentally proven, an interaction between carbohydrates and XlnR was already proposed by Hasper et al. [40]. The same was suggested for the ortholog Xyr1 in *Trichoderma reesei* and also for the *Saccharomyces cerevisiae* Gal4 itself [41].

Two regulatory mechanisms for the transcription dynamics were proposed: i) activation by *xlnR*, and ii) regulation by CreA (main effect ~3 to 4 h). Significant de-repression is observed in the Mt for some target genes, especially using high Xyl concentrations. Overall, the proposed model to a large extent explains the dynamics of the XlnR regulon concerning the opposing effects of XlnR activation and CreA repression. It also provides an explanation for the larger expression rates achieved with the 1 mM stimulus compared to the 50 mM. We have demonstrated that the same model structure can be used to describe the dynamics of most XlnR targets in experiments involving both the Mt and Wt – especially at low Xyl inducing concentration.

Analysis of our model in relation to the data indicates that some regulatory elements or mechanisms controlling the XlnR regulon are missing. For example post-translational modifications of XlnR and CreA have not been considered. Moreover, additional, yet unstudied TFs (both, activators and repressors) are likely involved in regulation of at least part of the XlnR regulon in an indirect or direct manner. The possible auto-regulatory influence of XlnR might play a role in the regulatory network. Finally, the mechanisms of the loss of function of CreA in the mutant may need some further study.

However, the need for re-parameterization between Wt and Mt still leaves room for further structural improvements. Some of the patterns observed in the higher order dynamics using 50 mM Xyl for induction still remain a challenge to be accurately described; however, in the future, uncovering more regulatory components through experiments should enable further refining of the proposed model in our work. Unraveling the complexity in regulatory mechanism of the network is crucial for engineering strains with enhanced ability to produce PCWDE.

## Conclusions

In this work we investigate the dynamics of the XlnR regulon using mathematical modeling coupled with experimental data of gene expression profiles. We have shown that the frequent sampling of the high resolution TCD in our work revealed interesting dynamics in the data. These datasets are useful for network inference and mathematical modeling, particularly for the XlnR regulon that has not been extensively modeled before.

## Availability of supporting data

The data used for the modeling and supporting the results and conclusions of this article are included within the article and its additional files.

## Additional files

Additional file 1:
**Matlab codes.** The Matlab codes used for modeling the Mt. (ZIP 22 kb) Additional file 2:
**Matlab codes.** The Matlab codes used for modeling the Wt. (ZIP 19 kb) Additional file 3: Table S1. Parameter estimates and standard deviations for the Wt and Mt TCD. **Table S2.** Model goodness-of-fit *R* ^2^ estimates for the Mt and Wt TCD. (DOCX 24 kb) Additional file 4: Figure S1. Dynamic time warping clustering of expression profiles. **Figure S2.** Hill function plots for data obtained from the Mt using 1 mM Xyl. **Figure S3.** Hill function plots for data obtained from the Mt using 50 mM Xyl. **Figure S4.** Hill function plots for TCD obtained from the Wt using 1 mM Xyl. **Figure S5.** Hill function plots for TCD obtained from the Wt using 50 mM Xyl. (ZIP 1939 kb) Additional file 5:
**Data for Mt.** Data used for the modeling. (ZIP 3 kb) Additional file 6:
**Data for Wt.** Data used for the modeling. (ZIP 7 kb)

## Abbreviations

RT-qPCR: reverse transcription quantitative polymerase chain reaction
TF: transcription factor
PCWDE: plant cell wall degrading enzymes
ODE: ordinary differential equation
TCD: time course data
HPLC: high performance liquid chromatography
URS: upstream repressing sequence
UAS: upstream activating sequence
Mt: Mutant
Wt: wild type
Xyl: D-xylose
