# Supplementary material for: Quantitative modeling and analytic assessment of the transcription dynamics of the XlnR regulon in Aspergillus niger
Source: BMC Syst Biol. 2016 Jan 29;10:13. doi: 10.1186/s12918-016-0257-4 (PMC4731903; doi:10.1186/s12918-016-0257-4)
Supplement: Additional file 1: — Matlab codes. The Matlab codes used for modeling the Mt. (ZIP 22 kb) [file 12918_2016_257_MOESM1_ESM.zip › Description_Codes_Mt.docx]

**Description of Matlab codes used for modeling with the Mt strain data:**

- **mtcallmodelfit2data.m:** Main file for calling the model optimization/parameter estimation run.
- **mtfitmodel1mmxyl.m:** Model fit to data when some parameters (least sensitive ones as described in the manuscript) are fixed.
- **mtlsqfit2data.m:** A function with the ode45 routine for integration during the model fit to data.
- **mtmodelfit2data1mm.m:** Model fit to data using the optimal sets of estimated parameters from the model fitting.
- **mtplotHillfunctions.m:** Plot the Hill functions related to the models using the optimal parameters obtained from the model fit to data.
- **mtTranslationModel.m:** The model specifications with the translation process included.
- **shadedErrorBar.m:** A Matlab routine that creates an attractive shaded error region rather than discrete bars.

We recommend that all the files be put in one folder with the associated data when running the codes.
