# Supplementary material for: Quantitative modeling and analytic assessment of the transcription dynamics of the XlnR regulon in Aspergillus niger
Source: BMC Syst Biol. 2016 Jan 29;10:13. doi: 10.1186/s12918-016-0257-4 (PMC4731903; doi:10.1186/s12918-016-0257-4)
Supplement: Additional file 3: Table S1. — Parameter estimates and standard deviations for the Wt and Mt TCD. Table S2. Model goodness-of-fit R 2 estimates for the Mt and Wt TCD. (DOCX 24 kb) [file 12918_2016_257_MOESM3_ESM.docx]

**Table S1. Parameter estimates and standard deviations for the Wt and Mt TCD.** Four parameters were fixed at ${\hat{\bar{K}}}_{\mathrm{on}}=14.957$, ${\hat{\bar{K}}}_{\mathrm{off}}=75.541$, ${\hat{\bar{k}}}_{1}=21.455$ and ${\hat{\bar{k}}}_{2}=20.065$ for estimations in both the Wt and Mt. Some parameters were estimated with good precision (low CVs) and others not. The parameter estimates $\hat{K}_{i2,\mathrm{Wt}}$ and $\hat{k}_{id}$ in italics are noticeably poor since their transcription profiles showed no evidence of CreA repression and mRNA degradation for the 5 h experimental window. The ratio $\hat{\lambda}_{i}={\hat{k}_{is,\mathrm{Mt}}}/{\hat{k}_{is,\mathrm{Wt}}}>1$ for all the 7 target genes for which both the Wt and Mt data was available. To calculate $\hat{k}_{is,\mathrm{Mt}}$ and $\hat{K}_{i2,\mathrm{Mt}}$ for the Mt, the estimates $\hat{K}_{i1}$ and $\hat{k}_{id}$ from the Wt were fixed.

| Parameter estimates and standard deviations | | | | | | |
| --- | --- | --- | --- | --- | --- | --- |
| Strain | Wild type (Wt) | | | | Mutant (Mt) | |
| Gene | $\hat{k}_{is,\mathrm{Wt}}\pm\mathrm{SD}\left( \hat{k}_{is,\mathrm{Wt}} \right)$ | $\hat{K}_{i1}\pm\mathrm{SD}\left( \hat{K}_{i1} \right)$ | $\hat{K}_{i2,\mathrm{Wt}}\pm\mathrm{SD}\left( \hat{K}_{i2,\mathrm{Wt}} \right)$ | $\hat{k}_{id}\pm\mathrm{SD}\left( \hat{k}_{id} \right)$ | $\hat{k}_{is,\mathrm{Mt}}\pm\mathrm{SD}\left( \hat{k}_{is,\mathrm{Mt}} \right)$ | $\hat{K}_{i2,\mathrm{Mt}}\pm\mathrm{SD}\left( \hat{K}_{i2,\mathrm{Mt}} \right)$ |
| *xlnB* | 438.780$\pm$83.210 | 0.125$\pm$0.026 | 34.112$\pm$2.729 | 3.725$\pm$0.815 | 1663.600$\pm$138.880 | 29.118$\pm$1.934 |
| *xlnD* | 30.000$\pm$10.103 | 0.111$\pm$0.079 | 31.410$\pm$7.339 | 1.965$\pm$1.235 | 123.350$\pm$9.583 | 44.096$\pm$2.084 |
| *xyrA* | 320.000$\pm$74.618 | 0.083$\pm$0.052 | 34.558$\pm$6.250 | 1.692$\pm$1.004 | 378.600$\pm$18.374 | 39.356$\pm$1.250 |
| *axeA* | 214.650$\pm$56.299 | 0.141$\pm$0.040 | 37.702$\pm$4.865 | 2.817$\pm$0.687 | 1300.600$\pm$118.520 | 35.188$\pm$1.760 |
| *axhA* | 85.478$\pm$25.027 | 0.258$\pm$0.033 | 61.402$\pm$6.682 | 3.897$\pm$1.020 | 2845.700$\pm$495.150 | 17.380$\pm$5.989 |
| *aguA* | 118.430$\pm$22.141 | 0.092$\pm$0.013 | 55.332$\pm$5.008 | 3.277$\pm$0.661 | 1217.300$\pm$84.977 | 39.269$\pm$1.722 |
| *faeA* | 227.490$\pm$43.985 | 0.220$\pm$0.025 | 42.949$\pm$3.093 | 2.881$\pm$0.472 | 5323.2$\pm$342.060 | 26.611$\pm$1.054 |
| *xlnC* | 98.376$\pm$27.324 | 0.109$\pm$0.015 | 68.656$\pm$10.995 | 4.269$\pm$1.118 | - | - |
| *eglA* | 61.593$\pm$17.674 | 0.075$\pm$0.011 | 100.000$\pm$52.081 | 4.443$\pm$1.249 | - | - |
| *eglC* | 30.000$\pm$25.305 | 0.415$\pm$0.193 | 50.000$\pm$15.908 | 1.000$\pm$0.469 | - | - |
| *talB* | 29.199$\pm$3.139 | 0.109$\pm$0.010 | 50.000$\pm$2.622 | 2.684$\pm$0.338 | - | - |
| *xdhA* | 100.000$\pm$27.637 | 0.101$\pm$0.015 | 100.000$\pm$59.788 | 3.251$\pm$0.797 | - | - |
| *ladA* | 200.000$\pm$432.560 | 0.281$\pm$0.473 | 35.000$\pm$31.971 | 2.023$\pm$2.920 | - | - |
| *estA* | 4.659$\pm$0.859 | 0.031$\pm$0.008 | 70.000$\pm$9.676 | 1.000$\pm$0.263 | - | - |
| *abfB* | *12.000*$\pm$*191.530* | *0.222*$\pm$*2.772* | *10.000*$\pm$*169.370* | *1.000*$\pm$*3.919* | - | - |
| *bglA* | 24.466$\pm$2.943 | 0.061$\pm$0.024 | 37.522$\pm$3.522 | 1.231$\pm$0.464 | - | - |
| *xkiA* | 128.520$\pm$104.540 | 0.116$\pm$0.028 | 80.000$\pm$36.370 | 6.170$\pm$4.873 | - | - |
| *eglB* | 3.366$\pm$1.436 | 0.010$\pm$0.001 | 60.564$\pm$3.807 | 1.625$\pm$0.774 | - | - |
| *cbhA* | *0.437*$\pm$*0.179* | *0.101*$\pm$*0.038* | *100.000*$\pm$*160.300* | *1.0e-5*$\pm$*0.034* | - | - |
| *cbhB* | *0.107*$\pm$*0.122* | *0.154*$\pm$*0.172* | *200.000*$\pm$*1.3*$e+4$ | *2.7e-4*$\pm$*8.000* | - | - |
| *lacA* | *66.219*$\pm$*17.480* | *0.077*$\pm$*0.012* | *200.000*$\pm$*1.6*$e+4$ | *3.403*$\pm$*0.852* | - | - |
| *aglB* | *0.762*$\pm$*0.149* | *0.039*$\pm$*0.012* | *200.000*$\pm$*1.6*$e+4$ | *0.320*$\pm$*0.099* | - | - |

**Table S2. Model goodness-of-fit** $R^{2}$ **estimates for the Mt and Wt TCD**. Here $R_{1}^{2}$ and $R_{50}^{2}$ are the $R^{2}$ - statistics for the 1 and 50 mM Xyl datasets; $R_{\mathrm{comb}}^{2}$ is the $R^{2}$ statistic for the fit on the combined datasets for a given gene. Here, “-” represents unavailable data.

| Strain | Wild type (Wt): $R^{2}$– statistic | | | Mutant (Mt): $R^{2}$– statistic | | |
| --- | --- | --- | --- | --- | --- | --- |
| Gene | $R_{1}^{2}$ | $R_{50}^{2}$ | $R_{\mathrm{comb}}^{2}$ | $R_{50}^{2}$ | $R_{1}^{2}$ | $R_{\mathrm{comb}}^{2}$ |
| *xlnB* | 0.942 | 0.169 | 0.556 | 0.794 | 0.631 | 0.712 |
| *xlnD* | 0.798 | 0.505 | 0.652 | 0.923 | 0.667 | 0.795 |
| *xyrA* | 0.673 | 0.496 | 0.584 | 0.977 | 0.649 | 0.813 |
| *axeA* | 0.889 | 0.578 | 0.734 | 0.826 | 0.762 | 0.794 |
| *axhA* | 0.755 | 0.693 | 0.724 | 0.335 | 0.600 | 0.467 |
| *aguA* | 0.873 | 0.685 | 0.779 | 0.874 | 0.872 | 0.873 |
| *faeA* | 0.949 | 0.821 | 0.886 | 0.963 | 0.178 | 0.570 |
| *xlnC* | 0.873 | 0.583 | 0.728 | - | - | - |
| *eglA* | 0.836 | 0.502 | 0.669 | - | - | - |
| *eglC* | 0.226 | 0.584 | 0.405 | - | - | - |
| *talB* | 0.942 | 0.844 | 0.893 | - | - | - |
| *xdhA* | 0.912 | 0.529 | 0.720 | - | - | - |
| *ladA* | 0.873 | 0.279 | 0.576 | - | - | - |
| *estA* | 0.126 | 0.659 | 0.393 | - | - | - |
| *abfB* | 0.300 | 0.128 | 0.214 | - | - | - |
| *bglA* | 0.732 | 0.816 | 0.774 | - | - | - |
| *xkiA* | 0.809 | 0.188 | 0.498 | - | - | - |
| *eglB* | 0.263 | 0.127 | 0.195 | - | - | - |
| *cbhA* | 0.711 | 0.882 | 0.797 | - | - | - |
| *cbhB* | 0.065 | 0.160 | 0.113 | - | - | - |
| *lacA* | 0.874 | 0.544 | 0.709 | - | - | - |
| *aglB* | 0.015 | 0.563 | 0.289 | - | - | - |
