# Supplementary material for: Quantitative modeling and analytic assessment of the transcription dynamics of the XlnR regulon in Aspergillus niger
Source: BMC Syst Biol. 2016 Jan 29;10:13. doi: 10.1186/s12918-016-0257-4 (PMC4731903; doi:10.1186/s12918-016-0257-4)
Supplement: Additional file 4: Figure S1. — Dynamic time warping clustering of expression profiles. Figure S2. Hill function plots for data obtained from the Mt using 1 mM Xyl. Figure S3. Hill function plots for data obtained from the Mt using 50 mM Xyl. Figure S4. Hill function plots for TCD obtained from the Wt using 1 mM Xyl. Figure S5. Hill function plots for TCD obtained from the Wt using 50 mM Xyl. (ZIP 1939 kb) [file 12918_2016_257_MOESM4_ESM.zip › Supplementary_Figures.docx]

Supplementary Figures

**Fig. S1. Dynamic time warping clustering of expression profiles.** A and B: Clustering of gene expression profiles from Mt strain using 1 or 50 mM Xyl, respectively. C and D: Clustering of gene expression profiles from Wt strain using 1 or 50 mM Xyl, respectively.

**Fig. S2. Hill function plots for data obtained from the Mt using 1 mM Xyl.** A-G: Plot of regulation Hill functions for 7 target genes, the plots contain combined functions corresponding to the transcription data. The solid and dotted lines correspond to the Xyl and CreA Hill functions $\psi_{\mathrm{Xyl}}$ and $\psi_{\mathrm{CreA}}$, respectively. H: *xlnR* transcript levels.

**Fig. S3. Hill function plots for data obtained from the Mt using 50 mM Xyl.** A-G: Plot of regulation Hill functions for 7 target genes, the plots contain combined functions corresponding to the transcription data. The solid and dotted lines correspond to the Xyl and CreA Hill functions $\psi_{\mathrm{Xyl}}$ and $\psi_{\mathrm{CreA}}$, respectively. H: transcript levels of *xlnR*.

**Fig. S4. Hill function plots for TCD obtained from the Wt using 1 mM Xyl.** A-V: Plot of regulation Hill functions for the target genes, the plots contain combined functions corresponding to the transcription data. The solid and dotted lines correspond to the Xyl and CreA Hill functions $\psi_{\mathrm{Xyl}}$ and $\psi_{\mathrm{CreA}}$, respectively. W: *xlnR* transcript levels.

**Fig. S5. Hill function plots for TCD obtained from the Wt using 50 mM Xyl.** A-V: Plot of regulation Hill functions for the target genes, the plots contain combined functions corresponding to the transcription data. The solid and dotted lines correspond to the Xyl and CreA Hill functions $\psi_{\mathrm{Xyl}}$ and $\psi_{\mathrm{CreA}}$, respectively. W: *xlnR* transcript levels.
