# Supplementary figures and images for: Quantitative modeling and analytic assessment of the transcription dynamics of the XlnR regulon in Aspergillus niger
Source: BMC Syst Biol. 2016 Jan 29;10:13. doi: 10.1186/s12918-016-0257-4 (PMC4731903; doi:10.1186/s12918-016-0257-4)

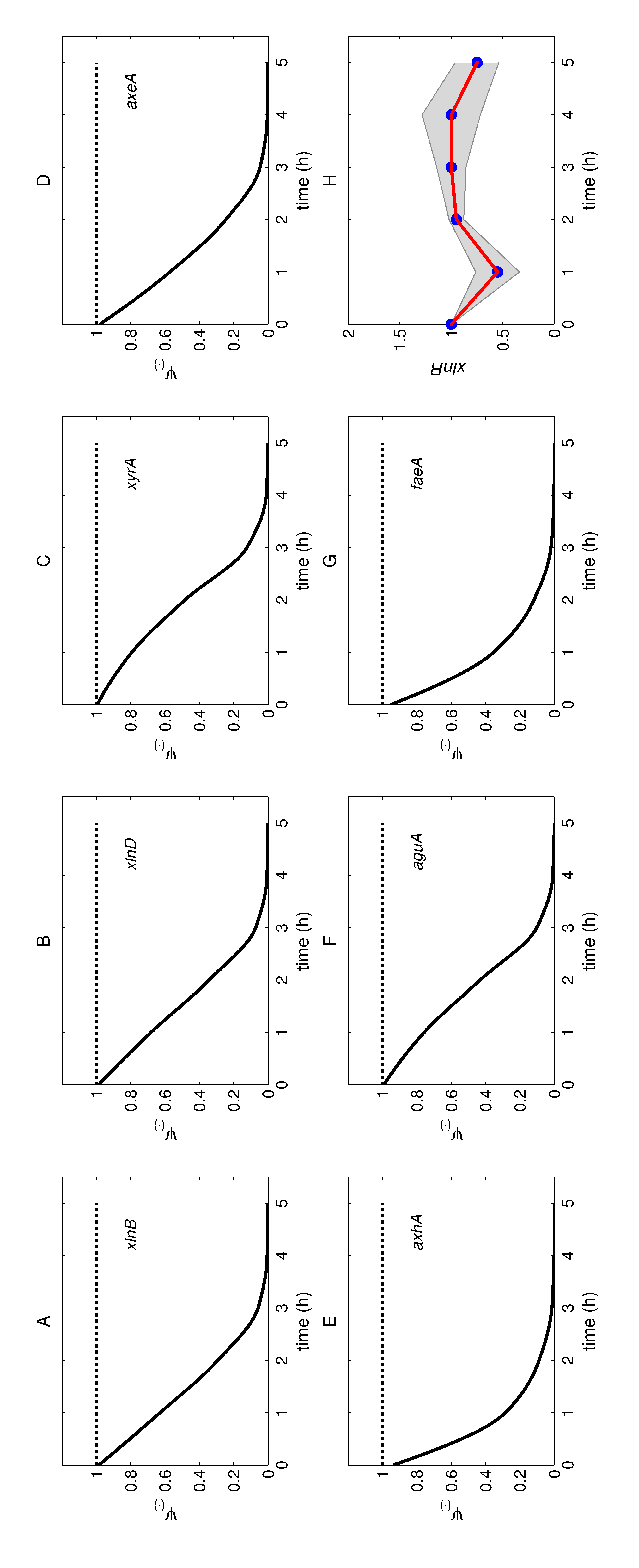

Supplement: Additional file 4: Figure S1. — Dynamic time warping clustering of expression profiles. Figure S2. Hill function plots for data obtained from the Mt using 1 mM Xyl. Figure S3. Hill function plots for data obtained from the Mt using 50 mM Xyl. Figure S4. Hill function plots for TCD obtained from the Wt using 1 mM Xyl. Figure S5. Hill function plots for TCD obtained from the Wt using 50 mM Xyl. (ZIP 1939 kb) [file 12918_2016_257_MOESM4_ESM.zip › FigureS2.tiff]

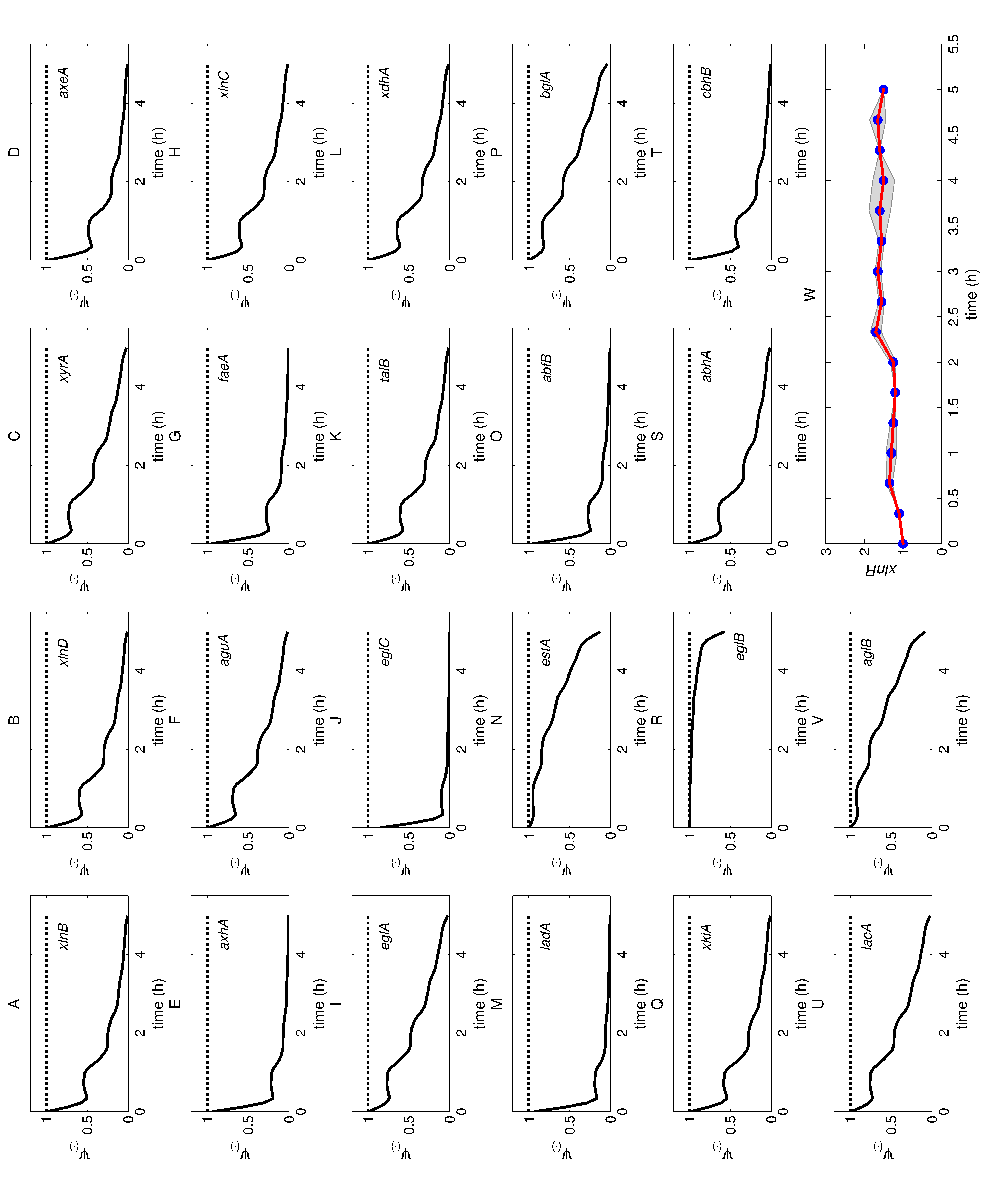

Supplement: Additional file 4: Figure S1. — Dynamic time warping clustering of expression profiles. Figure S2. Hill function plots for data obtained from the Mt using 1 mM Xyl. Figure S3. Hill function plots for data obtained from the Mt using 50 mM Xyl. Figure S4. Hill function plots for TCD obtained from the Wt using 1 mM Xyl. Figure S5. Hill function plots for TCD obtained from the Wt using 50 mM Xyl. (ZIP 1939 kb) [file 12918_2016_257_MOESM4_ESM.zip › FigureS4.tiff]

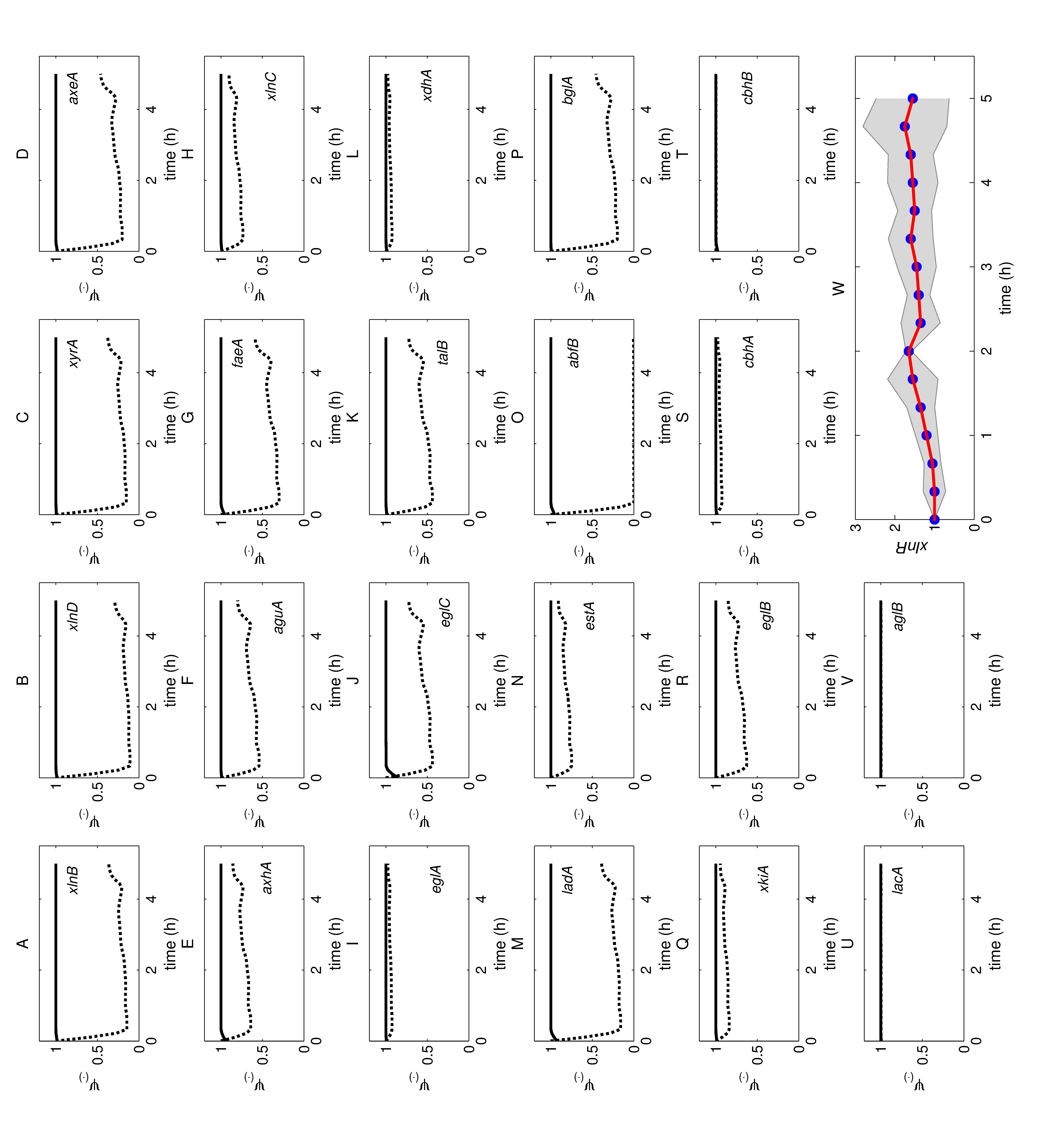

Supplement: Additional file 4: Figure S1. — Dynamic time warping clustering of expression profiles. Figure S2. Hill function plots for data obtained from the Mt using 1 mM Xyl. Figure S3. Hill function plots for data obtained from the Mt using 50 mM Xyl. Figure S4. Hill function plots for TCD obtained from the Wt using 1 mM Xyl. Figure S5. Hill function plots for TCD obtained from the Wt using 50 mM Xyl. (ZIP 1939 kb) [file 12918_2016_257_MOESM4_ESM.zip › FigureS5.tiff]

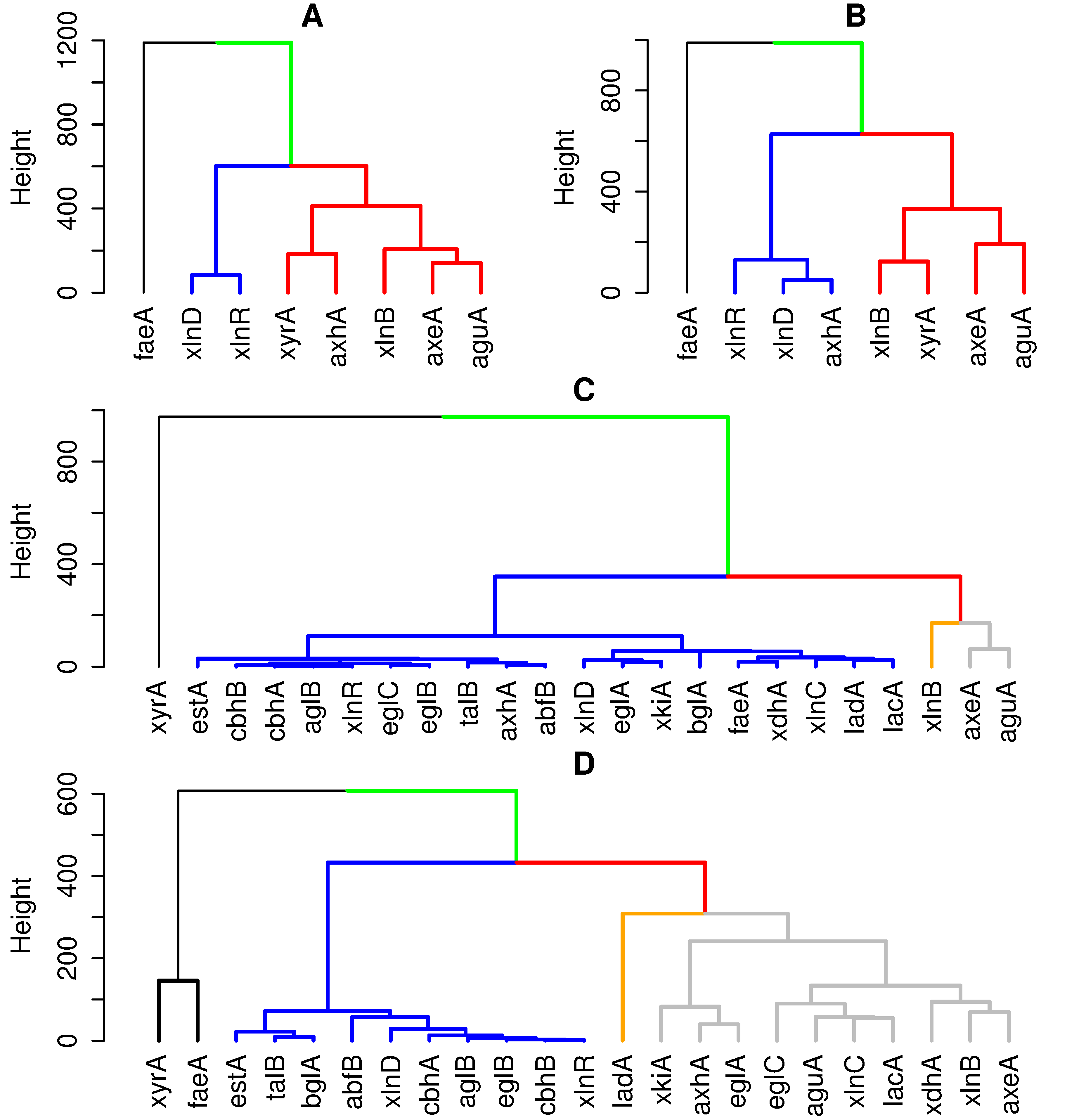

Supplement: Additional file 4: Figure S1. — Dynamic time warping clustering of expression profiles. Figure S2. Hill function plots for data obtained from the Mt using 1 mM Xyl. Figure S3. Hill function plots for data obtained from the Mt using 50 mM Xyl. Figure S4. Hill function plots for TCD obtained from the Wt using 1 mM Xyl. Figure S5. Hill function plots for TCD obtained from the Wt using 50 mM Xyl. (ZIP 1939 kb) [file 12918_2016_257_MOESM4_ESM.zip › FigureS1.tiff]

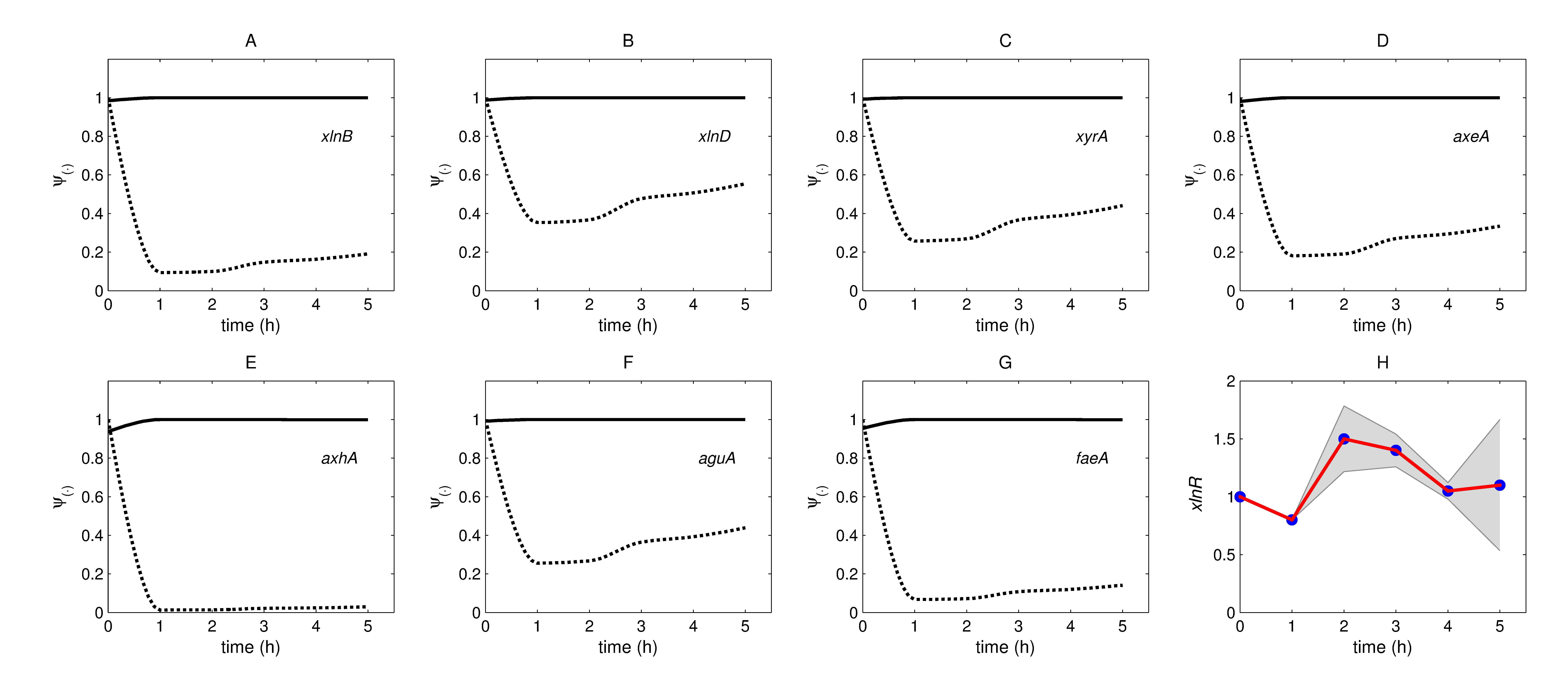

Supplement: Additional file 4: Figure S1. — Dynamic time warping clustering of expression profiles. Figure S2. Hill function plots for data obtained from the Mt using 1 mM Xyl. Figure S3. Hill function plots for data obtained from the Mt using 50 mM Xyl. Figure S4. Hill function plots for TCD obtained from the Wt using 1 mM Xyl. Figure S5. Hill function plots for TCD obtained from the Wt using 50 mM Xyl. (ZIP 1939 kb) [file 12918_2016_257_MOESM4_ESM.zip › FigureS3.tiff]
